# Anterior Cingulate Cortex Causally Supports Meta-Learning

**DOI:** 10.1101/2024.06.12.598723

**Published:** 2024-06-13

**Authors:** Robert Louis Treuting, Kianoush Banaie Boroujeni, Charles Grimes Gerrity, Paul Tiesinga, Thilo Womelsdorf

**Affiliations:** 1 Department of Biomedical Engineering, Vanderbilt University, Nashville, TN 37240; 2 Vanderbilt Brain Institute, Nashville, TN 372404; 3 Department of Psychology, Vanderbilt University, Nashville, TN 37240; 4 Princeton Neuroscience Institute, Princeton University, Princeton, NJ 08540; 5 Department of Electrical and Computer Engineering, Vanderbilt University, Nashville, TN 37240; 6 Donders Institute for Brain, Cognition and Behaviour, Radboud University Nijmegen, Nijmegen 6525 EN, Netherlands.

**Keywords:** Cognitive flexibility, micro-stimulation, nonhuman primate, task difficulty, motivation, reinforcement learning, working memory, prediction error

## Abstract

In dynamic environments with volatile rewards the anterior cingulate cortex (ACC) is believed to determine whether a visual object is relevant and should be chosen. The ACC may achieve this by integrating reward information over time to estimate which objects are worth to explore and which objects should be avoided. Such a higher-order meta-awareness about which objects should be explored predicts that the ACC causally contributes to choices when the reward values of objects are unknown and must be inferred from ongoing exploration. We tested this suggestion in nonhuman primates using a learning task that varied the number of object features that could be relevant, and by controlling the motivational value of choosing objects. During learning the ACC was transiently micro-stimulated when subjects foveated the to-be-chosen stimulus. We found that stimulation selectively impaired learning when feature uncertainty and motivational value of choices were high, which was linked to a deficit in using reward outcomes for feature-specific credit assignment. Application of an adaptive reinforcement learning model confirmed a primary deficit in weighting prediction errors that led to a meta-learning impairment to adaptively increase exploration during learning and to an impaired use of working memory to support learning. These findings provide causal evidence that the reward history traces in ACC are essential for meta-adjusting the exploration-exploitation balance and the strength of working memory of object values during adaptive behavior.

## Short Summary

The anterior cingulate cortex encodes object-specific reward histories, but how this information contributes to behavior has remained unclear. This paper shows that micro-stimulating the anterior cingulate when subjects fixate multidimensional objects whose values are not yet known impairs learning their value. Micro-stimulation reduced prediction error weighting and impaired the meta-learning adjustment of the exploration-exploitation trade-off and the use of working memory of object values during periods of uncertainty.

## Introduction

Lesions of the anterior cingulate cortex (ACC) impair the ability to choose objects with the highest values ^1,2^. This deficit is particularly pronounced when the ACC is disrupted in volatile environments where the value of objects probabilistically vary over time ^2–4^, but impairments are also apparent in learning and set shifting paradigms that require shifting choices between objects that have otherwise fixed reward associations ^1,5–7^. For all these paradigms, ACC disruption does not result in persevering with the same non-optimal choices, but rather in a difficulty sustaining attending to and choosing objects once they are rewarded. It has been suggested that one reason for this pattern of deficits after ACC disruption is an impaired integration of reward information over time to estimate the value of currently available objects ^8,9^ and to guide the next choice to the option with the highest predicted value ^3,10^. A key unresolved question of this account of the ACC is how an integration of reward and choice histories translates into guiding choices of specific objects.

One solution to translating reward information over time into choices is to track rewards explicitly in a working memory of object values. Support for a role of the ACC in this process comes from a disruption study that found the ACC mediates choosing counterfactual stimuli that are not available as options for a current choice but which would be the best valued alternative option in future trials based on their past values ^3^. This result indicated that object values are maintained even for unavailable options and that the ACC plays a role translating them into a choice. A similar conclusion comes from studies that tracked how subjects have to infer the value of visual features in environments with multiple features ^7,11^. In these multidimensional environments, the use of working memory of recently rewarded objects is a model mechanism that can speed up learning beyond the use of slower reinforcement learning mechanisms that rely on learning value estimates using prediction errors ^11^. These insights complement the role of short-term memory of rewarded actions to support learning complex sensori-motor association ^12–16^. This behavioral and modeling evidence supports a role of working memory in learning. The key open question we address in this study is whether neural circuits in ACC support this type of working memory of relevant objects.

A second way to use reward history to improve choices is to adjust the exploration-exploitation balance. Exploiting the same option is beneficial in stable environments and the ACC does play only limited roles in environments with no changes in reward values ^2,17,18^. However, exploring choice options becomes necessary when previously rewarded options cease to provide reward or reward values of options are uncertain. A role of the ACC in guiding exploration during such periods of uncertainty has been suggested in learning experiments ^14,19^, in studies showing reduced exploratory fixational sampling of objects after ACC disruption ^7^ and in neuronal activity correlating with information seeking behavior ^20^. These insights suggest the ACC mediates exploratory behavior when reward history signals an increase in uncertainty about which options are most reward predictive ^8,21^.

To test for a causal role of the ACC in mediating exploration and working memory for object values we devised a task that increased the demands on exploratory sampling and on working memory by varying the number of visual features subjects needed to evaluate during learning. In addition, we varied the motivational value of choosing objects by varying the gains and losses for correct and erroneous choices. We found that micro-stimulating the ACC during learning of feature values systematically disrupts learning when the motivational value is high and the feature space is large, thereby reducing the ability to sustain choices of recently rewarded objects. A meta-reinforcement learning model ^11^ suggested that stimulation selectively affected the learning process by impairing the working memory of recently rewarded objects and the ability to adaptively increase exploration of choice options when reward uncertainty was high.

## Results

**Figure 1.**
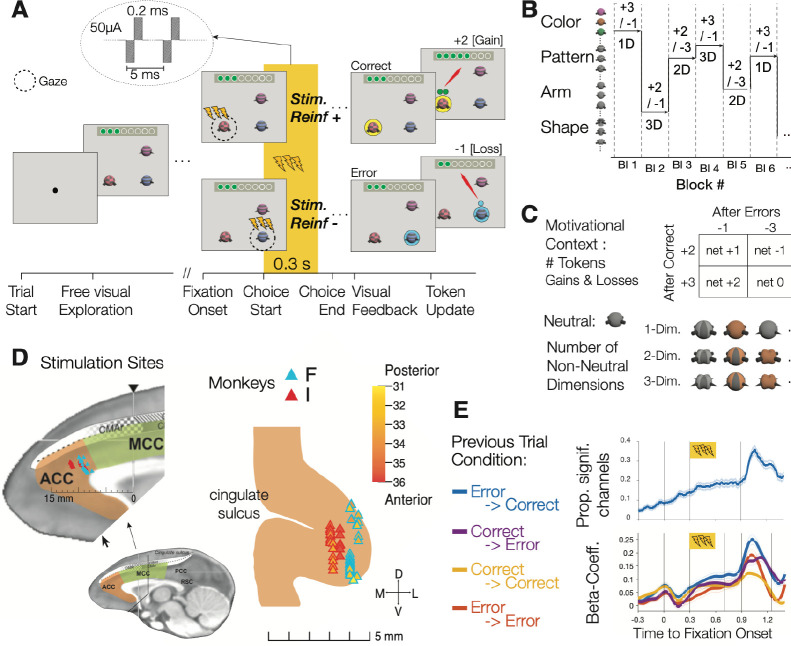
Task paradigm, stimulation locations and neural signatures of previous trial effects. (**A**) On each trial monkeys freely explored three stimuli until they fixated on one stimulus for longer than 0.3 s, which indicated they committed choosing that stimulus. Electrical (or sham) stimulation was delivered 0.3–0.6 s after this choice-fixation either when the positive reinforced object was chosen (StimReinf+) or when a negative reinforced stimulus was chosen (StimReinf−). Stimulation was only delivered during learning new feature-reward associations, i.e. prior to reaching a 70% performance criterion. 0.9 s after the choice-fixation onset a halo behind the chosen object provided feedback (yellow/blue for correct/error choices), followed 0.2 s later by auditory feedback and revealing the tokens gained or lost. (**B**) The task switched the rewarded feature (y-axis) in blocks of 30–50 trials. (**C**) Blocks randomly varied the motivational context from gaining +2 or +3 for choosing the object with the rewarded feature, and the loss of −1 or −3 tokens otherwise. Blocks also varied randomly whether features from one, two, or three feature dimensions of the objects changed from trial to trial (1D, 2D, and 3D conditions). (**D**) Stimulation locations in the ACC for monkeys F / I (blue / red) in the reference frame of Procyk et al, 2016 (left) and shown on a coronal view (right). Coloring indicates the anterior-posterior distance to the interaural line. (**E**) Proportion of channels with MUA activity significantly modulated by the outcome of previous trials (top), and beta coefficients (bottom) of a regression model predicting the outcome of the current trial using previous trial outcomes as predictors.

We applied electrical micro-stimulation to the fundus of the anterior cingulate cortex (area 24c) in 24 sessions in each of two monkeys (total of 48 sessions). Brief 0.3 s stimulation was delivered during learning of feature-reward rules when monkeys fixated an object to indicate a choice of that object, where stimulation either was administered when the rewarded target object (*Stim-Reinf+*) or the non-rewarded (*Stim-Reinf−*) was chosen. (Figure 1A). In each trial of the learning task monkeys were shown three objects and explored them with fixational eye movements until they chose an object by sustaining fixation of an object for 0.7 s Choices were followed by visual and auditory feedback and by the addition of tokens for correct responses or the subtraction of already attained tokens for incorrect responses. Tokens were green coin symbols in a token bar. Accumulating eight tokens resulted in fluid reward. The rewarded target feature remained constant for a block of 30–50 trials before it switched to a new feature that was not shown before (Figure 1B). Blocks pseudo-randomly varied the motivational context (+2 or +3 tokens gained for correct choices; loss of −1 or −3 tokens for errors), and the learning difficulty. Difficulty levels corresponded to the number of visual features that distinguished objects from trial-to-trial, increasing from features of one, two, to three feature dimensions (1D, 2D, and 3D learning conditions) (Figure 1C). Monkeys F and I on average completed 33.5 and 36 blocks per session.

Micro-stimulation focused on the cognitive subfield of the ACC that has prominent connectivity to the lateral prefrontal cortex and executive function networks ^22–24^ (Figure 1D). Neural activity in this ACC site codes for values of covertly attended stimuli ^25^, values of choice options ^26,27^, and prediction errors of visual features ^28,29^. One hallmark of neuronal responses of this ACC site is they change when previous trials’ outcomes were unfavorable, unexpected, or when they trigger a behavioral change of choice strategies ^28–31^. We confirmed in neural recordings that multiunit activity at the micro-stimulation sites showed previous-trial modulation with larger increases of activity during a correct choice that followed a previous erroneous choice compared to correct choices that followed other correct choices (Figure 1E). These findings indicate that activity at the ACC sites used for stimulation varied depending on behavioral adjustment at the time when subjects fixated the object they choose, which we used as stimulation time window.

### Micro-stimulation impairs learning when difficulty and motivational saliency are high

**Figure 2.**
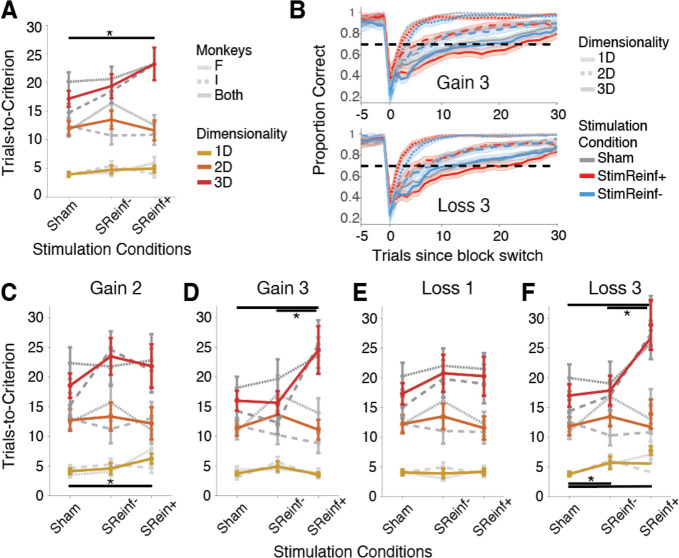
Anterior cingulate cortex stimulation impairs learning in difficult and motivationally challenging conditions. (**A**) Average number of trials to reach 70% performance criterion (y-axis) for the 1D/2D/3D dimensionality conditions for the sham, StimReinf+, StimReinf− stimulation conditions. Grey lines show individual monkeys. Horizontal bar shows significantly slower learning in StimReinf+ than Sham condition for high dimensionality blocks (FDR-corrected t-test, p = 0.007). (**B**) Average Learning curves for stimulation and dimensionality conditions and dimensionalities in blocks leading to gaining 3 tokens for correct choices (upper) or loosing 3 tokens for incorrect choices. Error bars are SEs. For learning curves in other conditions, see **Fig. S1**. (**C-F**) Same format as A for blocks where correct trials led to gains of +2 tokens (C), +3 tokens (D), and when erroneous choices led to a loss of −1 tokens (E) and −3 tokens (F). Error bars are SEM. P-values < 0.05 indicated with *.

Without micro-stimulation (*Sham condition*) monkeys successfully learned feature-reward rules at >70% correct in the 1D/2D/3D conditions after an average of 4/12/20 trials (monkey F) and 4/12/15 trials (monkey I) (Figure 2A). Micro-stimulation significantly slowed learning when it was delivered in the difficult 3D condition while subjects chose the correct, positively reinforced stimulus (*StimReinf*+ stimulation condition) (ANOVA Stimulation condition x dimensionality interaction: F = 3.2, p = 0.0125). Monkeys reached the >70% learning criterion on average after 23.24 trials within a block compared to 17.16 trials in the *Sham* condition in the 3D condition (FDR-corrected t-test, p = 0.0070); trials-to-criterion for monkey F: 23.26 versus 20.12; monkey I: 23.22 versus 14.76) (Figure 2A). Stimulation did not change the overall likelihood that the learning criterion was reached within a block (**Figure S1A**). Closer inspection of the results showed that the learning impairment in the difficult condition varied with motivational saliency, (ANOVA 3-way interaction: stim. condition x dimensionality x gain conditions, F = 2.42, p = 0.0465; for loss: F = 1.39, p = 0.2337). Both monkeys were impaired in the 3D condition in blocks when the incentive for correct choices was highest (gains of +3 tokens; FDR-corrected t-tests, *StimReinf*+ vs *Sham* and *StimReinf*+ vs *StimReinf*−: p = 0.0025 and p = 0.0078), and when the punishment for incorrect choices was largest (loss of −3 tokens; FDR-corrected t-tests, *StimReinf*+ vs *Sham* and *StimReinf*+ vs *StimReinf*−: p = 0.0057 and p = 0.0308) (Figure 2C–F; **Figure S1E-H**). Micro-stimulation also slowed down learning in the easier (1D) condition when incentives were low and punishments were high but this was less consistent across monkeys (Figure 2D,F).

Micro-stimulation selectively impaired learning without affecting plateau performance once learning was completed (**Figure S1B-D**), reaction times of choosing objects during learning (**Figure S2A**), or viewing times of objects prior to making a choice in a given trial during learning (**Figure S2B-E**). Impaired learning was evident across the experimental session (**Figure S3**).

To discern whether the micro-stimulation effect depended on how strong the stimulation pulses modulated neural activity at the stimulation site, we extracted the post-stimulation multiunit activity peaks in the stimulation and sham conditions for individual sessions. We found no correlation between the strength of the neuronal stimulation effect on activity and the behavioral stimulation effect on learning speed across conditions (**Figure S4**).

### Micro-stimulation during correct choices interferes with credit assignment

**Figure 3.**
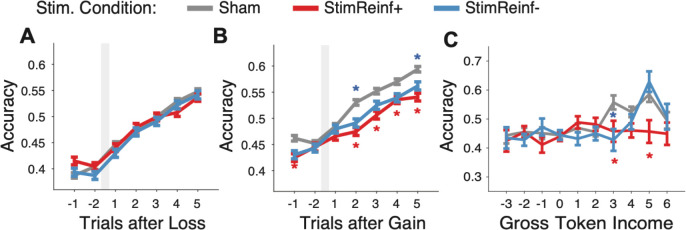
Stimulation impairs behavioral adjustment after correct trials and after accumulating tokens. (**A**) Accuracy in trials before and after trials with an erroneous choice (grey vertical bar) during learning (E_n_ analysis) for the Sham (grey), StimReinf+ (red), and StimReinf− (blue) condition. (**B**) Same format as A for trials before and after trials with a correct choice (C_n_ analysis). Analysis is restricted to trials during learning, i.e. before the learning criterion was reached. Red and blue stars denote a difference at p < 0.05 (permutation test) for the StimReinf+ and StimReinf− condition compared to Sham. (**C**) Same color schema as A,B showing accuracy (y-axis) as a function of the accumulated ‘gross token income’ calculated from the preceding two trials. The analysis considered trials up until the learning criterion was reached in a block. The stars indicate FDR corrected significance at p < 0.05 based on a permutation test. Individual monkey results are shown in **Figure S5**.

The impaired learning was evident particularly in the *StimReinf*+ condition, which suggests that stimulation interfered with positive credit assignment for correctly chosen features, but not when negative credit assignment was needed after error trials. We tested this suggestion by analyzing how subjects improved their performance in trials following correct (rewarded) compared to incorrect (unrewarded) outcomes. We found that neither *StimReinf*−, nor *StimReinf*+ altered the trial-by-trial adjustment after an error outcome (Figure 3A), but that with *StimReinf*+ stimulation in correct trials subjects were impaired in improving performance compared with *Sham* stimulation, resulting in reduced performance in the 2^nd^ - 5^th^ trial following a correct choice (permutation test, p < 0.05; for trials 2, 3, 4 and 5 after correct trials: p values were p = 0.0003, p = 0.0039, p = 0.0372, and p = 0.0006 respectively, FDR corrected) (Figure 3B). Both monkeys showed this deficit in improving performance after *StimReinf*+ stimulation (**Figure S5A**,**B**), suggesting that *StimReinf*+ stimulation interferes with assigning positive value to specific features of the chosen stimulus. Stimulation during erroneous choices in the *StimReinf*− condition on average reduced performance 2 and 5 trials after correct choices (permutation test, p < 0.05; p = 0.0124 and p = 0.0408, FDR corrected) (Figure 3B), but this was not consistent across monkeys (**Figure S5B**).

We confirmed the impaired use of positive outcomes for learning by analyzing performance improvements as a function of the number of tokens subjects had accumulated over the last two trials ^7^. When subjects attained three or more tokens in the past trials in the *Sham* condition, they more likely continued to be correct, indicating a motivational benefit of having accumulated tokens and getting closer to collecting all eight tokens and cashing them out for primary fluid reward. Such a motivational effect was absent in the *StimReinf*+ condition, confirming an impaired use of positive feedback to improve performance (Figure 3C).

### ACC micro-stimulation impairs adjusting exploration during learning

**Figure 4.**
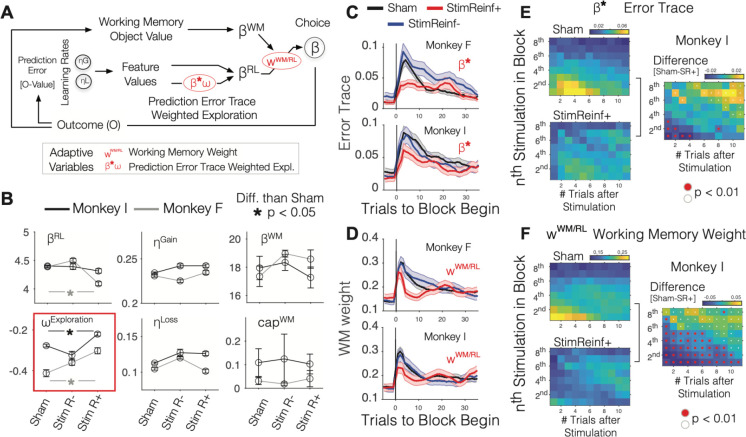
The Meta reinforcement learning model and its static and dynamic variables during ACC stimulation. (**A**) Model schematic. The RL component calculates prediction errors to estimate feature values, while the WM component directly uses outcomes to update the WM representation. The behavioral choice is a combination of the object value in WM and the feature values in the RL. The weighting of the WM content (w ^WM/RL^) and the prediction-error-trace (β*) are adaptive variables that change trial-by-trial. w ^WM/RL^ changes according to how reliable WM content is, and β* changes according to the history of recent prediction errors. (**B**) Parameter values of the model fit of the Sham, StimReinf+, and StimReinf− conditions for each monkey. See **Figure S6** for more details. (**C**) The prediction-error-trace (β*) is calculated for trials relative to the block begin in each condition for monkey F (upper) and I (lower). Error shading denote SE. (**D**) Same format as C for the WM weight (w^WM/RL^). (**E**) The average prediction-error-trace (β*) in trials t_+1_, t_+2 …_ t_+12_ (x-axis) after the 1^st^, 2^nd^ … to 8^th^ stimulation within a block in the Sham (upper left) and StimReinf+ (lower left) condition for monkey I. The prediction-error-trace difference (Sham minus StimReinf+) is shown on the right. Red/white dots denote significantly reduced/enhanced prediction-error-trace in the StimReinf+ condition (at p<0.01 , randomization test). (**F**) Same format as E for the WM weight (w ^WM/RL^). See **Figure S7** for results of each monkey.

Micro-stimulation impaired learning feature-reward rules. This effect could be due to reduced learning rates, or to impairments in other cognitive processes supporting efficient learning ^11,32,33^. We evaluated three separable processes that together were previously shown to account for learning in multidimensional environments ^11^: (*i*) a meta-learning mechanism that adaptively increases exploration of objects during learning when prediction errors accumulate ^11,14,34^, (*ii*) valence-specific updating of expected values with separate learning rates for gains and losses ^11,35^, and (*iii*) an adaptive working memory (WM) mechanism that maintains recently rewarded objects in WM to speed-up learning ^12,16,33^ (Figure 4A, see Methods). Fitting the monkeys’ behavior with the model showed that most static model variables, including the learning rates, did not vary between the *Sham*, *StimReinf*+, and *StimReinf*− conditions (Figure 4B). The only exception was an increase of the exploration weight ($\omega^{\mathrm{Exploration}}$, increased values reflect increased exploitation). The model parameter $\omega^{\mathrm{Exploration}}$ determines how meta-aware subjects adjust exploration when prediction errors accumulate by weighting the prediction-error-trace parameter $\left(\beta^{*}\right)$ (Figure 4A, see Methods). Micro-stimulation in the *StimReinf*+ condition increased $\omega^{\mathrm{Exploration}}$, i.e. increased exploitation over exploration, compared to the *Sham* condition in both subjects (*StimReinf*+ vs *Sham*, t-test, p<0.05 for monkeys I and F) (Figure 4B). In the *Sham* condition, the prediction-error-trace $\left(\beta^{*}\right)$ sharply increased at the beginning of a learning block, but not in the *StimReinf*+ condition (Figure 4C). This result shows that micro-stimulation in the *StimReinf*+ reduced the build-up of a history of prediction errors, captured in the $\beta^{*}$ parameter. The increase of the meta-learning weight $\omega^{\mathrm{Exploration}}$ and reduced modulation of $\beta^{*}$ with *StimReinf*+ stimulation is consistent with the scenario that the meta-learning weight ($\omega^{\mathrm{Exploration}}$) increased in an attempt to compensate for the blunted prediction error trace to minimize the negative consequences of increased uncertainty about feature values during learning.

### ACC micro-stimulation reduces the use of working memory of recent object values

ACC micro-stimulation also impaired the second dynamic learning mechanism that adaptively weighs how strong choices are influenced by the content of WM (Figure 4D). The model chooses an object based on combining the choice probability of a WM component and the choice probability of an RL component with the weighting parameter *w*^*WM/RL*^ (see Methods). The WM is weighted larger the better it predicts rewarded choices compared to the prediction using the expected value of the RL component ^11^. Using WM is a fast, ‘one-shot’ learning mechanism, that allows repeating a rewarded choice without waiting for the slower, incremental updating of feature values in the RL component of the model ^15,16^. Consistent with the use of WM for learning, the WM-to-RL weight parameter *w*^*WM/RL*^ increased at the beginning of a learning block in the *Sham* and *StimReinf*− conditions, but *w*^*WM/RL*^ did not rise as high and dropped more abruptly with *StimReinf*+ stimulation (Figure 4D). Thus, ACC micro-stimulation impaired using the faster WM mechanism as a strategy for learning.

### Stimulation has immediate consequences on performance

The micro-stimulation effects on the dynamic weighting of exploration and WM were evident immediately after the first stimulation and did not gradually built up (Figure 4E,F; **Figure S7**). We tested the time course of the effects by calculating the prediction-error-trace (*β*^∗^) and the WM weight (*w*^*WM/RL*^) for trials (t_+1_, t_+2,…_ t_+12_) that followed the first stimulation (*stimTrial*_*1*_), the second stimulation (*stimTrial*_*2*_), and up to the eights stimulation (*stimTrial*_*8*_) within each block. We found that after *Sham* stimulation the prediction-error-trace rose immediately, peaked 3–5 trials after the first sham-stimulation trial, and gradually normalized after about 10 trials (Figure 4E). This contrasted to *StimReinf*+ trials for which stimulation caused only a delayed and shallower increase of the prediction-error-trace in post-stimulation trials (Figure 4E). Comparing *StimReinf*+ to Sham confirmed that already the first stimulation resulted in a reduced prediction-error-trace that lasted for 4 (monkey I, **Figure S7A**) and 2 (monkey F, **Figure S7B**) subsequent trials (p<0.01, for each monkey, randomization test). For one subject (monkey I) weighting of the prediction-errortrace was reduced after each of the first four stimulations, while it continued to be lower for the other subject (monkey F). Similarly, immediate post-stimulation effects were evident for the WM weight, which already rose after the first stimulation trial in the *Sham*, but not in the *StimReinf*+ condition (Figure 4F). For the first six stimulation trials in both subjects the weighting of WM remained lower in the *StimReinf*+ compared to *Sham* condition for more than six trials (**Figure S7C**,**D**). These findings indicate that stimulation reduced the role of WM content for performance throughout the early learning period of a block.

## Discussion

We have shown that micro-stimulating the ACC when subjects fixated an object that is correctly chosen (*StimReinf*+ condition) impaired inferring the relevance of features of that object during learning. Learning was impaired when objects varied in multiple feature dimensions (*3D condition*) and the motivational saliency of the choice was high (losing 3 instead of 1 token, and gaining 3 instead of 2 tokens) (Figure 2). The learning deficit could be traced back to an inability to use positive choice outcomes to improve performance (Figure 3). Behavioral learning is governed by latent variables that are not easily observed but need to be estimated from the sequence of behavioral choices. We modeled various latent learning mechanisms and found that micro-stimulation affected predominantly adaptive meta-learning parameters as opposed to static reinforcement learning variables. Micro-stimulation impaired adjusting exploration and the use of working memory of object values to inform choices during learning (Figure 4). Taken together, these findings show that the fundus of the ACC causally supports the flexible meta-adjustment of performance.

### ACC causally supports meta-learning.

We observed apparent learning deficits when two conditions were met: when the motivational salience of a choice was high (high gains or losses) and when the credit assignment about feature values was difficult (in the 3D condition). This pattern of result shows that the ACC fundus causally supports the multiplexing of motivational and cognitive functions and is consistent with two major accounts of ACC function: Consistent with a motivational effort-control account ^36^, the ACC causally supported learning when the motivational saliency of choices is high by either anticipating a high motivational benefit (gaining 3 vs 2) or a high potential punishment (loss of 3 vs 1). And consistent with a cognitive account, the ACC causally guided searching for choice options when outcome uncertainty was high (with 3 vs 2 or 1 varying feature dimensions) ^9,37^. Importantly, the multiplexing of motivation and cognitive variables is consistent with the ACC’s rich direct anatomical connectivity to subcortical sites that directly affect motivation, including dopaminergic neurons, and that mediate attentiveness and cognitive control, including cholinergic and norepinephrinergic nuclei ^38,39^. This specific anatomical connectivity has been used as a key argument for suggesting that the ACC adaptively boost cognitive control when task demands are high ^40^. Our results provide direct causal support for this ‘boosting’ function of the ACC. Importantly, our modeling results suggest that the theoretically proposed function of the ACC to ‘boost cognitive control’ ^41^ is realized (1) by enhancing exploration, which reduces perseverative responding, and (2) by enabling working memory of rewarded objects to influence choices during learning. Thus, our results suggest that the ACC causally contributes to flexible cognitive control by adaptively enhancing exploration and working memory during learning, ultimately increasing learning speed through meta-learning adjustments of learning-relevant cognitive processes ^42^.

### ACC supports feature-specific credit assignment.

Conceptualizing the ACC as mediating metalearning is consistent with neurophysiological evidence that neuronal subpopulations in the ACC encodes different types of uncertainty. Uncertain outcomes during instrumental learning and Pavlovian conditioning triggers ACC activity reflecting surprise, prediction errors, and uncertainty about appetitive and aversive outcomes ^20,27,29,31,43,44^. For one group of neurons these uncertainty signals carry information about the specific features that preceded the uncertain outcome ^29^. Such a feature-specific prediction-error signal is a powerful teaching signal for credit assignment because it informs the subject which specific feature it should or should not choose in future encounters. Our study suggests that micro-stimulation interfered with these feature-specific prediction error signals when there was ambiguity about which feature dimension should be used for credit assignment in the 3D condition. This conclusion is consistent with the model results that micro-stimulation reduced the weighting of prediction errors during learning.

Taken together, the present study suggests that 0.3 s brief, gaze-contingent stimulation of the ACC during adaptive decision making interferes with credit assignment of the chosen features. Analysis of the latent processes that impaired prediction errors provide causal evidence of the ACC’s role as a meta-learner whose activation is necessary to flexibly adapt exploration and using working memory as a strategy to efficiently infer the relevance of visual objects in our environment.

## Methods

### Ethics Statement.

All animal and experimental procedures were in accordance with the National Institutes of Health Guide for the Care and Use of Laboratory Animals, the Society for Neuroscience Guidelines and Policies, and approved by the Vanderbilt University Institutional Animal Care and Use Committee (M1700198–01).

### Experimental Procedures.

Two adult male macaque monkeys performed the experiments (monkey F, 13 years of age, 11.3 kg, and monkey I, 11 years of age, 11.0 kg). Behavior and electrical stimulation triggers were controlled using the Multi-Task Unified Suite for Experiments (M-USE) ^45^. The animals were trained on the feature-reward rule learning task in cage-based touchscreen Kiosk Testing Station ^46^ and stimulation experiments proceeded in sound attenuating experimental booths with subject’s head position fixed, facing a 21” LCD screen at a distance of 63 cm from their eyes to the screen center. For each stimulation experiment, a tungsten stimulation electrode (FHC Inc., Bowdoin, ME) was lowered through a guide tube at a pre-determined location in a custom recording/stimulation chamber implanted over the left hemisphere. The stimulation electrodes were lowered into the ACC and stopped once sustained spiking activity was noticeable and constant.

### Task paradigm.

The feature-reward rule learning task required learning through trial-and-error which feature of multidimensional objects is associated with reward. Objects were 3D rendered Quaddles ^47^ that could vary features in 1,2, or 3 feature dimensions within a block. The feature dimensions were body shape, arm type, color, and body pattern with 9–11 feature per dimensions (e.g. oblong, pyramidal, spherical, etc. for body shapes). In each block, only one feature value was associated with token reward. The feature that was rewarded, i.e. the feature-reward rule, stayed constant for 30–50 trials, then switched randomly to another feature in a new block. Monkeys initiated a trial by fixating a black circle for 0.5 s, which triggered after a 0.3 s delay the presentation of three objects randomly positioned at three of the four corners of a virtual square-grid spanning 10.5 cm with 5° eccentricity relative to the screen center. Objects extended over ~3cm on the screen corresponding to ~2.5° degrees of visual angle. The animals had up to 5 s to choose one object by maintaining gaze at an object for >0.7 s Choosing the correct object was followed by a yellow halo around the stimulus as visual feedback (500 ms), an auditory tone, and either 2 or 3 tokens (green circles) added to the token bar (*G2* and *G3* conditions). Choosing an object without the rewarded target feature was followed by a blue halo around the selected objects, a lower-pitched auditory feedback, and for the loss conditions, the presentation of a grey ‘loss’ token(s) that traveled to the token bar where one already obtained token was removed. In different conditions either 1 or 3 tokens were lost after incorrect choices (*L1* and *L3* conditions). The timing of the feedback was identical for gain and loss conditions. In each session, monkeys were presented with up to 36 separate learning blocks, each with a unique feature-reward rule.

Across all 48 experimental sessions, monkeys F/I completed on average 33.5/36 blocks per session. For each experimental session, a unique set of objects was defined by randomly selecting three dimensions and three features per dimension (e.g., 3 body shapes: oblong, pyramidal and ellipsoid; 3 arm types: upward pointy, straight blunt, downward flared; 3 patterns: checkerboard, horizontal striped, vertical sinusoidal) ^47^. Based on this feature set three different task conditions were defined: One task condition contained objects that differed in features of only one dimension, while features of the other dimensions were identical, i.e., the object body shapes were oblong, pyramidal, and ellipsoid, but all objects had blunt straight arms and uniform grey color (‘1D’ condition). A second task condition defined objects that varied features in two dimensions (‘2D’ condition), and a third task condition defined objects that varied in features of three dimensions (‘3D’ condition). Learning which feature is rewarded is systematically more demanding with objects varying in more feature dimensions ^7^.

### Experimental design.

Each session was composed of 36 feature-reward rule blocks that pseudo-varied four motivational conditions and three cognitive demand conditions. Cognitive demand varied the number of object features that varied from trial to trial with features varying in one, two, or three dimensions (conditions 1D, 2D, or 3D). The motivational conditions varied the amount of tokens gained for correct choices to be 2 or 3 (G2 and G3 conditions) and the amount of tokens subtracted (lost) from incorrect choices were either −1 or −3 (L1 and L3 conditions) as introduced in prior studies ^48^. Subjects had to earn 8 tokens to completely fill a token bar on top of the screen. Each block began with an asset of 3 tokens. This starting asset ensured that subjects lost tokens already after the first incorrect trial in a block and thereby recognized the block’s loss condition. Across the 36 blocks all combinations of motivational conditions (+2/−1, +3/−1, +2/−3, +3/−3) and cognitive conditions (1D, 2D, 3D) were pseudo randomly assigned to blocks in equal number.

Each session contained 15 blocks with electrical stimulation, 15 sham stimulation blocks, and 6 initial blocks without stimulation. A session was designated either a *StimReinf*+ or a *StimReinf*− session in which *Sham* and one of the stimulation conditions (*StimReinf*+ or *StimReinf*−) alternated starting after the sixth block. A session ended either when all 36 blocks were completed, or when a monkey stopped initiating trials after 90 minutes.

### Monitoring and analysis of gaze.

Gaze was monitored with the binocular infrared eye-tracker at 600 Hz sampling rate (Spectrum, Tobii). Gaze was calibrated before the task began with a 9-point eye-tracker calibration routine. Object fixations were reconstructed using a robust gaze classification algorithm described in ^49^ . The duration of object fixations was analyzed for objects fixated prior to the last fixation onto the object that the subjects choose by maintaining fixation for >0.3 s onto that object.

### Electrical micro-stimulation.

Electrical stimulation was delivered with the Intan Stimulation Controller (RHS 2000, Intan Technologies, LLC), starting 300 ms after monkeys continuously fixated an object and lasted for 300 ms. Choice fixations lasted >700 ms ensuring that the electrical stimulation was administered only while the chosen object was fixated. Monkey’s fixation durations had a bimodal distribution with the fixations indicating the sampling of information prior to making a choice lasting 150–200 ms on average in the 1D,2D, and 3D conditions, and the last fixation that monkeys used to indicate a choice lasting on average 800 ms (see Suppl. Figure S7 in ^7^). The 300 ms lasting 200 Hz electrical stimulation pulse train consisted of 60 biphasic 50mA pulses, with a 0.2 ms cathodal current injection followed immediately by a 0.2 ms anodal current, and a 5ms interval between onsets of these 0.4 ms lasting pulses (Figure 1A).

### Stimulation-triggered multi-unit activity and its relation to learning performance.

Prior to starting stimulation within an experimental session, we ensured through visual and auditory inspection of neural activity that the stimulation electrode was in the grey matter at sites in which neurons showed spiking activity. Offline, we quantified how strong multi-unit activity at the stimulation electrode was modulated by electrical stimulation and tested whether the modulation of stimulation-triggered multi-unit activation (MUA) was predictive of behavioral effects of stimulation. To this end, we extracted continuous multi-unit activity using a 0.5–3 kHz bandpass filter, followed by rectification, and 200 Hz low-pass filtering of the recorded signal before and after the 0.3 s stimulation period. We removed outlier trials reflective of artifacts with the interquartile methods and for each session normalized the data to a −0.3–0 sec pre-stimulation baseline period. To quantify the stimulation triggered effect, we extracted the maximum response within a 0.65–1.3 s post-stimulation window in the stimulation and sham conditions, and calculated a *MUA Index* as the normalized difference (*Stim* − *Sham*)/(*Stim* + *Sham*) of that maximum response. The *MUA Index* ranges from −1 to +1 with positive values reflecting stronger MUA activity in the stimulation condition compared to the sham condition. For the same sessions we also quantified the learning speed (trials-to-criterion) and calculated a *Performance Index* (*Stim* − *Sham*)/(*Stim* + *Sham*). We statistically analyzed the relation of the MUA Index and the Performance Index for those experimental conditions in which stimulation affected learning using Pearson correlations.

### Reconstruction of anatomical stimulation locations.

We reconstructed the recording and stimulation sites using the software 3D slicer (http://www.slicer.org, version 4.11). We first co-registered pre-MRI (or post-MRI) with post-CT scans for both monkeys, segmented the recording chamber, then reformatted the image space to reference the chamber space. We recorded the x,y,z (z being the depth of the electrode) coordinate the stimulation electrode tips relative to the chamber to reproduce the penetration depth for each stimulation electrode. The reconstructed electrode tip site was labeled on the MRI images, where monkey F’s stimulation sites were overlayed onto monkey I’s image to have a unified image of stimulation sites and in addition to monkey I having better image quality (Figure 1D).

### Multi-unit analysis of previous-trial effects at stimulation sites.

Electrical micro-stimulation was performed at sites within the ACC that showed stronger signals when the monkey chose correctly an object after an error trial than when it continued correct performance after correct trials, which indicates that these sites encode prediction error signals that are possible teaching signals for improving future performance. We evaluated the ACC sites in a separate set of recording experiments in which two monkeys performed the same experimental task as during the stimulation experiment (13/12 recording sessions). One of these monkeys (F) was used for the stimulation experiment. In these recording-only sessions neural activity was recorded with 64-channel linear silicon probes (Neuronexus, 50 μm interelectrode distance) using Open-Ephys (open-ephys.org) and the Intan Recording System (RHD2000, Intan Technologies, LLC). Neuronal wideband recordings were notch filtered (60, 120, and 180 Hz) with a filter bandwidth of 2 to remove power noise and its harmonics, and re-referenced using median referencing across channels. Continuous multi-unit activity (MUA) was quantified by 750–5000 Hz bandpass filtering, rectifying, 300 Hz low pass filtering, and down-sampling to 1000 Hz. Individual trials were extracted from trial initiation until the token and reward feedback was given with additional 0.5 s padding. Trials were aligned to the time of the decision, and MUA smoothed using a gaussian window of 0.2 s We then used the mean activity during the baseline period 0.5 sec at the beginning of the trial and prior to the onset of the stimuli to detrend the data channel-wise. We then determined whether a recording channel showed task modulated neuronal activity with a regression model that predicted MUA activity every 25 msec using as factors trial outcome (correct vs error), reward outcomes (gain and loss of tokens), learning status (before vs. after trial-to-criterion was reached), stimulus dimensionality (1-,2-, and 3 dimensional objects), and previous trial outcome (correct vs error). We removed channels that showed no task modulation for any of these task variables in at least three consecutive time windows which affected on average 0.5 channels per probe.

We analyzed whether neuronal sites showed previous-trial dependent activity using linear regression of MUA activity applied for 0.2 s time windows shifted every 0.05 s relative to the time of the choice for trials depending on the previous trials’ outcome. We analyzed correct trials depending on whether they were preceded by an error trial (EC sequence) or a correct trial (CC sequence), and we analyzed error trials depending on whether they were preceded by an error (EE) or a correct trial (CE). We calculated the proportion of channels that showed a significant regression using the full regression model with all variables and analyzed the beta coefficients for those channels showing a significant modulation of either the EC, CC, EE, or CE conditions.

### Block-level analysis of learning and plateau performance.

We estimated the efficiency of learning by calculating the trials required to reach criterion performance as the first trial within a block at which animals reached 70% accuracy over the following 12 trials. To compare the trials-to-criterion between conditions we used two-way Student’s T tests with FDR corrected (alpha = 0.05) p-values. If monkeys did not reach this learning criterion during a block, we calculated the trial at which the learning criterion would have been reached by using a linear regression of the last 12 trials in the block to find the predicted trials needed to reach criterion. We counted these blocks as being unlearned to identify that the proportion of learned blocks did not differentiate the experimental conditions (**Figure S1A**). For the condition with *StimReinf*− stimulation, we excluded blocks with less than 2 active stimulation trials (i.e. with less than 2 errors). We applied two-way Student’s T tests with FDR corrected (alpha = 0.05) p-values to compare plateau accuracy of the animals. Plateau performance was indexed as the average accuracy across trials once the learning criterion was reached (**Figure S1C-D**).

### Analysis of Variance (ANOVA) on experimental conditions on Learning.

N-way ANOVAs were used to determine if there were significant group differences on learning (measured by trials-to-criterion for the block) between the stimulation conditions (*Sham, StimReinf+*, and *StimReinf*−), the cognitive conditions (*1D, 2D,* and *3D*), the Gain condition (*G2* and *G3*), and the Loss condition (*L1* and *L3*). We reported the significant (p < 0.05) main and interaction effects, as either 2-way (stimulation and cognitive conditions) or 3-way (stimulation, cognitive, and Gain or Loss conditions).

### Analysis of choice reaction times.

Stimulation might have an influence on the reaction time from display onset to the time when an object is fixated. We analyzed choice reaction times separately for correct trials in which the subjects made only one saccade and chose the first object they fixated and trials in which they made a second and third object fixation. The distribution of choice reaction times was done separately for the *sham*, *StimReinf*+, and *StimReinf*− conditions from the first block in which stimulation began (the sixth block onwards). Choice reaction time distributions for trials with one, two, and three object fixations were compared with a Wilcoxon rank sum test with FDR corrected (alpha = 0.05) p-values (**Figure S2A**).

### Analysis of fixational sampling of objects prior to choice.

We quantified how extensive monkeys explored objects within trials prior to making a choice by calculating the duration of stable fixations on objects prior to the last fixation of an object that indicated it was chosen (**Figure S2B-E**). Fixational sampling was calculated for each trials relative to the start of the block.

### Temporal stability of stimulation effects on learning.

We subdivided the number of stimulation blocks into three equally sized sets of ten blocks (trials 7–16; 17–26; 27–36) and calculated the trials to learning criteria separately for the stimulation conditions and dimensionality conditions in order to discern whether the effect of stimulation on learning varied over time within a session (**Figure S3**). We used the two-way Student’s T test with FDR corrected (alpha = 0.05) p-values to test for significance between the learning periods.

### Analysis of previous trial effects on accuracy: Behavioral adjustment following errors, correct choices, and variable accumulated tokens.

We quantified the accuracy (proportion correct choices) for those trials that were preceded by different types of behaviors separately for the *Sham*, *StimReinf*+, and *StimReinf*− conditions. These analyses were limited to trials during learning, i.e. prior to completion of learning and performed with data combined from both monkeys (Figure 3) and for each monkey separately (**Figure S5**). We calculated accuracy in trials relative to an erroneous choice (Figure 3A), relative to a correct choice (Figure 3B), and as a function of the number of tokens accumulated over the preceding two trials ^7^. The number of accumulated tokens indexes the motivational state of the monkeys and was calculated by taking the sum of earned tokens over the preceding two trials. Windows of three and four trials were tested and provided similar but noisier results. The main analysis was limited to the trials prior to completion of learning, but results were qualitatively similar when all trials were considered.

To compare stimulation effects on previous trial dependent performance, we used permutation tests. For the post-error and post-correct performance, we randomly shuffle, without replacement, the stimulation condition labels (*Sham*, *StimReinf*+, *StimReinf*−) of the trial outcome for each n^th^ trial (n=1–5 trails following the outcome), for 10000 iterations to establish a randomized sampling distribution of the mean difference between groups. We then calculated if the probability of a finding a value greater than the observed mean difference per stimulation condition following an outcome; the null hypothesis being that accuracy following a given trial outcome is not different across stimulation conditions. The p-value calculated is the probability that the difference between the groups mean being greater than the observed mean for each n^th^ trial. P-values were FDR corrected (alpha = 0.05) across post outcome trials.

The randomization approach for the accumulated token analysis randomly shuffled, without replacement, the stimulation condition labels (*Sham*, *StimReinf*+, *StimReinf*−) of the trial outcome for each number of accumulated tokens (from −3 to +6 tokens). This was done for 10000 iterations to establish a randomized sampling distribution of the mean difference between groups. We then calculated if the probability of finding a value greater than the observed mean difference per stimulation condition; the null hypothesis being that accuracy following a given number of accumulated tokens is not different between stimulation conditions. P-values were FDR corrected (alpha = 0.05) across post outcome trials.

### Behavioral modeling combining reinforcement learning, working memory and adaptive exploration.

We modeled the choices of the monkeys with a recently proposed reinforcement learning model ^11^ that combines the classical mechanism of reinforcement learning (RL) of features values updated using reward prediction errors, an attentional mechanism that decays the values of non-chosen (non-attended) object features, a working memory mechanism that updated object values in a working memory buffer with the last rewarded object, and an adaptive mechanisms that adjusted the degree of exploration relative to exploitation depending on the recent history of negative prediction errors. This *adaptive RL-WM model* generates on every trial two hypothetical choices based on the RL and the WM component and arrives at the final choice by weighting the expected feature values (RL) and object values (WM) using a weighting parameter that is estimated by fitting the model to the data (Figure 4A). There are several static model mechanisms and two adaptive variables of the adaptive RL-WM model that vary trial-by-trial depending on the performance of the subjects. The model is described and validated in detail elsewhere ^11^ and briefly introduced next.

#### Updating expected feature values using prediction errors.

The first static mechanism of the reinforcement learning (RL) model component is the updating of expected feature values (V) using reward prediction errors (RPEs) scaled by learning rates. The RL component is a traditional Rescorla Wagner Q-learning model that estimates for each trial the expected Q values of objects. For each of the three objects presented on a given trial, the object value is calculated as the sum of the values of its features $\left(V^{F}\right)$, which are updated on every trial according to the reward prediction error (RPE) equal to $\left(R_{t}\;-\;V_{i,t}^{F}\right)$, where R is the rewarded (1) or nonrewarded (0) outcome of the trials multiplied by a learning rate $\eta_{t}$ resulting in the equation $V_{t+1}^{F}=V_{i,t}^{F}+\eta_{t}\left(R_{t}\;-\;V_{i,t}^{F}\right)$. The learning rate for error outcomes ($\eta_{t}=\eta_{\mathrm{Loss}}$) is constant, but differs from the learning rate for correct outcomes $\left(\eta_{t}=\eta_{\mathrm{Gain}}\right)$, similar to ^35^. The RL component also used a selective forgetting mechanism by decaying non-chosen (nc) and not-presented (np) features by $\omega_{nc,t}=\omega_{np,t}=\omega^{RL}$. This mechanism prioritizes learning from chosen object features, which are also those that were attended when making a choice.

#### Softmax choice mechanism.

The second mechanism implements choice probability based on expected values using softmax winner-take all selection. On any given trial, each object has a probability of being chosen from the RL component of the model according to a softmax selection among object values. The choice probability is calculated as $p_{i,t}^{RL}\;=\;\frac{exp\left(\beta_{t}^{RL}\sum_{j\in O_{i}}\;V_{j,t}^{F}\right)}{\sum_{k}\;exp\left(\beta_{t}^{RL}\sum_{j\in O_{k}}\;V_{j,t}^{F}\right)}$, which uses a $\beta^{RL}$ parameter (the inverse temperature that we interpret as exploration/exploitation weight) to weigh the sum of the values of all features $\left(V^{F}\right)$ of the presented set of objects $\left(O_{i}\right)$. At low $\beta^{RL}$ the choice is exploratory and less strictly determined by the value of objects, while at high values of $\beta^{RL}$ the choices follow the highest valued object and thus are exploitative.

#### Fast one-shot working memory.

The third mechanism is working memory of object values, which has static parameters for the capacity and the decay of working memory content. The working memory component of the adaptive RL-WM model holds an object in WM when it is chosen and rewarded, which is represented as an object’s WM value $\left(V_{i,t+1}^{WM}=1\right)$^15,16^. WM values $V^{WM}$ decay slowly by $\omega^{WM}$ which allows its maintenance over a few trials as long as the same object is not chosen without being rewarded. Indeed, when an object is chosen and not-rewarded, $V^{WM}$ is reset immediately to the default value $\frac{1}{n_{o}}$, here $n_{o}$ is the number of presented objects. This rapid updating of the WM after rewarded and unrewarded choices of an object reflects a ‘one-shot’ learning mechanism that does not use reward prediction errors (Figure 4A). WM content contributes to the choice according to a $\beta^{WM}$ parameter determining the choice probability $p^{WM}$ of object i on trial t through a softmax selection process $p_{i,t}^{WM}\;=\;\frac{exp\left(\beta^{WM}V_{i,t}^{WM}\right)}{\sum_{j}\;exp\left(\beta^{WM}V_{j,t}^{WM}\right)}$.

#### Adaptive changes of exploration-exploitation balance.

The first adaptive model mechanism adjusts the balance of exploration and exploitation by weighting the recent history of negative prediction errors. In the *adaptive RL-WM model*, a choice is predicted by a β^RL^ exploration parameter that is not fixed but varies over trials with the history of recent negative prediction errors. When subjects make successive errors during learning a new feature-reward association the β^RL^ value is reduced to increase exploratory choices among objects ^11,14^. The $\beta^{RL}$ value is lowered (causing more explorative choices) when the reward prediction errors $\left(\delta_{t}\right)$ across chosen features are negative and continued to be large in absolute value which occurs when subjects persevere on unrewarded features. $\beta^{RL}$ is increased after correct trials (more exploitative) when reward prediction errors start decreasing. The time-varying $\beta^{RL}$ is adjusted after each trial according to the prediction error trace $\beta_{t+1}^{*}=\beta_{t}^{*}+\alpha_{+}max\left(\delta_{t},0\right)+\alpha_{-}min\left(\delta_{t},0\right)$ where α− is 0.4 and α+=−0.6 and $\delta_{t}$ is the reward prediction error computed across all object features $\left(\delta_{t}=R_{t}-\right.\left.\frac{1}{\#\left(j\in O_{i}\right)}\sum_{j\in O_{i}}\;V_{j,t}^{F}\right)$, here i is index of the chosen object and t the trial as before. The prediction error trace is translated into an actual β value by weighting it with a (negative) exploration factor $\omega^{\mathrm{Exploration}}$ according to $\beta_{t}^{RL}=\frac{\beta_{m}}{1+exp\left(-\omega^{\mathrm{Exploration}}\left(\beta_{t}^{*}-\omega_{2}\right)\right)}$. The $\omega_{2}$ parameter was fixed to 0.5.

#### Adaptive changes of the weight of working memory relative to the reinforcement learning value.

The second adaptive mechanism is the trial-by-trial adjustment of the relative weight of the WM choice probabilities relative to the RL choice probabilities for a final choice. Previous work has shown that learning is better predicted when both, the RL and the WM component, influence choosing objects during learning ^11^. The relative influence of WM on choosing an object is determined by a weighting factor $w_{t}^{WM/RL}$ which combines the WM and RL choice probabilities into an integrated choice probability $\left(p^{IN}\right)$ calculated as $p_{i,t}^{IN}\;=\;w_{t}^{WM/RL}p_{i,t}^{WM}\;+\;(1\;-\;w_{t}^{WM/RL})p_{i,t}^{RL}$. A higher $w_{t}^{WM/RL}$ corresponds to a more prominent role of the WM component in determining the choice. We refer to $w_{t}^{WM/RL}$ as the working memory weight in Figure 4 and **Figure S7**. The model increases the WM influence (by increasing $w_{t}^{WM/RL}$) when it has sufficient capacity to hold the value of objects and when its choice probability is high relative to the choice probability of the RL component, which will occur particularly during the initial learning after a block switch when the expected values are only slowly adjusting while the working memory object value is updated immediately after a correct choice (Figure 4C).

### Estimating dynamic RL-WM model variables for each stimulation condition.

The static parameters and adaptive variables were estimated by fitting the adaptive RL-WM model to the behavioral data for each monkey and experimental condition by minimizing the Negative Log Likelihood (NLL) using the MATLAB (The Mathworks, Inc.) function *fminsearch* followed by calls to *fmincon*. Previous work established that the adaptive RL-WM model outperforms simpler models with less parameters in a similar learning task ^11^. We used the fitted model parameters to generate for each stimulation condition sequences of the prediction-error-trace ($\beta^{*}$) and the WM weight $\left(w^{WM/RL}\right)$, which are the two latent adaptive model variables that change from trial-to-trial. We estimated the variability of the model parameters, the prediction-error-trace and WM weight within experimental conditions using a Bootstrap approach that fit the behavioral data of each condition with random subsets of 80% of the blocks from that condition, generated the sequence of choice with the bootstrapped model parameter values, and extracted $\beta^{*}$ and $w^{WM/RL}$ from the model generated data. We visualize the mean and standard error of the mean of the prediction-error-trace and WM weight separately for each monkey for the *Sham, StimReinf−,* and *StimReinf*− condition in Figure 4C,D.

### Prediction error-trace and working memory weight following micro-stimulation trials.

We asked whether the prediction-error-trace ($\beta^{*}$) and the WM weight $\left(w^{WM/RL}\right)$ differed in the stimulation versus sham condition already after the initial trials with stimulation, or only later after multiple stimulation trials had accumulated within a block. To address this question, we extracted $\beta^{*}$ and $w^{WM/RL}$ separately for the first trial with stimulation or sham (stimTrial_t1_), the second sham or stimulation trial (stimTrial_t2_), and up to the eights sham or stimulation trial (stimTrial _t8_) within each block. For each of these stimTrial_t1–8_ we calculated the average $\beta^{*}$ and $w^{WM/RL}$ for each of eight trials that followed the stimTrial. This approach led to a two dimensional 8x12 matrix that spans stimTrial t_1–8_ on the y-axis and trials t_1–12_ that followed each of the stimTrial _t1–8_ on the x-axis (Figure 4E,F). We computed this matrix separately for each monkey for *Sham*, *StimReinf*+, and *StimReinf*− conditions. To test whether stimulation modulated this matrix relative to sham, we bootstrapped the model by fitting the adaptive WM-RL model twenty times, each time randomly subsampling 80% of the data. We then generated for each bootstrapped model the trial-by-trial changes of the prediction-error-trace (β*) and working memory weight $\left(\omega^{RL/WM}\right)$ and calculated the 95% confidence range for the bootstrapped sham control condition. This procedure was done for each cell of the 8 x 12 [stimTrial t_1–8_ ; trials t_1–12_ ] matrix. We then tested the null hypothesis that $\beta^{*}$ and $w^{WM/RL}$ from the sham and stimulation conditions were not different by testing whether the observed values in the *StimReinf*+ or *StimReinf*− condition were lower or higher than the lower or upper 95% confidence bound of the sham condition.

## Data Availability

Data and custom programming code for analysis and modeling is available upon reasonable request.
